# Predicting COVID-19 cases in Belo Horizonte—Brazil taking into account mobility and vaccination issues

**DOI:** 10.1371/journal.pone.0269515

**Published:** 2024-02-23

**Authors:** Eder Dias, Alexandre M. A. Diniz, Giovanna R. Souto, Henrique L. Guerra, Humberto Torres Marques-Neto, Simon Malinowski, Silvio Jamil F. Guimarães

**Affiliations:** 1 Computer Science Department, Pontifical Catholic University of Minas Gerais, Belo Horizonte, Minas Gerais, Brazil; 2 Geography Department, Pontifical Catholic University of Minas Gerais, Belo Horizonte, Minas Gerais, Brazil; 3 Dentistry Department, Pontifical Catholic University of Minas Gerais, Belo Horizonte, Minas Gerais, Brazil; 4 Medicine Department, Pontifical Catholic University of Minas Gerais, Belo Horizonte, Minas Gerais, Brazil; 5 Computer Science Department, University of Rennes 1, Rennes, France; East China Normal University, CHINA

## Abstract

The pandemic caused millions of deaths around the world and forced governments to take drastic measures to reduce the spread of Coronavirus. Understanding the impact of social distancing measures on urban mobility and the number of COVID-19 cases allows governments to change public policies according to the evolution of the pandemic and plan ahead. Given the increasing rates of vaccination worldwide, immunization data may also represent an important predictor of COVID-19 cases. This study investigates the impact of urban mobility and vaccination upon COVID-19 cases in Belo Horizonte, Brazil using Prophet and ARIMA models to predict future outcomes. The developed models generated projections fairly close to real numbers, and some inferences were drawn through experimentation. Brazil became the epicenter of the COVID-19 epidemic shortly after the first case was officially registered on February 25th, 2020. In response, several municipalities adopted lockdown (total or partial) measures to minimize the risk of new infections. Here, we propose prediction models which take into account mobility and vaccination data to predict new COVID-19 cases.

## Introduction

The outbreak of Coronavirus disease (COVID-19) caused by the SARS-CoV-2 led to serious health, economic, and social challenges worldwide. In terms of number of infections, in December 2021, 273 million cases and 5,3 million deaths have been officially reported worldwide [1]. Public health and social measures were implemented across the globe to suppress SARS-CoV-2 transmission and reduce mortality and morbidity from COVID-19, such as physical distancing measures that affect human mobility. The first case of COVID-19 confirmed in Brazil was on 25 February 2020 when a man from São Paulo tested positive for the virus. The first case in Belo Horizonte, Minas Gerais, Brazil was confirmed on March 21 2020, and the disease spread to the first quarter of 2021 in every federative unit of Brazil. Belo Horizonte is the sixth-largest city in Brazil, with a population of around 2.52 million commanding a metropolitan area with over 6 milion people, which is part of an important economic triangle in the Brazilian Southeast region. By June 16, 2021, 224,976 infections and 5,487 deaths had been notified in Belo Horizonte. Since the first reported case, public health policies and social distancing measures have been adopted to limit the number of infections and deaths. Social distancing [2] may be seen as is a primary and, in many cases, a necessary measure in combating virus dissemination.

Therefore, the impact of human mobility upon the dissemination of COVID-19 cases needs to be evaluated since it may help in stopping the virus dissemination. In this direction, several works were developed in order to study the impact of mobility [3–5]. Additionally, the development of indicators to estimate future cases of COVID-19 in urban centers could be used to suppress virus transmission such as SARS-CoV-2.

The development of indicators to estimate future cases of COVID-19 could help health authorities to make decisions about mobility restrictions and strategies of social distancing, that could delay the spread of the virus. Therefore, a good indicator of future cases could help achieving the best course of action and a better allocation of public heath resources. In the present study, by using mobility graph analysis it was possible to detect an increase in COVID-19 cases with increasing social mobility. However, changes that affect the behavior of the virus in the environment, such as mutations that make the virus more transmissible, vaccination that optimizes the immune response of the host making it less susceptible to infections, or periods of increased transmissibility that result in herd immunity are factors that may limit the development of indicators of future case based on mobility data.

In this context, the present study aims to evaluate the evolution of COVID-19 cases and deaths in Belo Horizonte, and the influence of local public health policy, such as the closing of establishments and commercial activities, upon the dissemination of COVID-19. Based on public health policy and human mobility indicators, we seek to estimate future COVID-19 cases. In terms of Brazil, other works have studied the behaviour of the spreading of COVID-19 cases between cities through mobile phone data [6], which may be seen as a mobility strategy as well.

The main contributions of this work can be summarized as follows: (i) study of the impact of mobility for spreading the COVID-19 infections; (ii) study of the effect of vaccination on transmission of SARS-CoV-2; (iii) proposition of three models based on time-series analysis for predicting new COVID-19 infections; and (iv) inclusion of the exogenous mobility variable, which is predicted by using Prophet framework, into the new cases prediction model.

## Materials and methods

### Ethics statement

This study was approved by the Research Ethics Committee from Pontifícia Universidade Católica de Minas Gerais, Brazil (4.359.790). Individual consent was not obtained because the data were analyzed anonymously.

### COVID-19 cases data

Belo Horizonte’s COVID-19 reported cases were gathered between March 20, 2020 and December 31, 2021. Data were retrieved from the online Epidemiological Bulletin of Belo Horizonte, available at: https://prefeitura.pbh.gov.br/saude/coronavirus, published by Belo Horizonte’s Municipal Health Secretariat, which brings daily updates on the official number of COVID-19 cases, transmission rates, occupation of ICU beds devoted to COVID-19 patients, ongoing vaccination rates, among other information. The dataset was constructed by manually extracting these daily data from the epidemiological bulletin. Data were subjected to three different analyses: one SARIMA model, using COVID-19 cases as the reference; and two ARIMA models with exogenous variables, one using Google Mobility data and another one combining mobility and vaccination variables.

**Fig 1:**
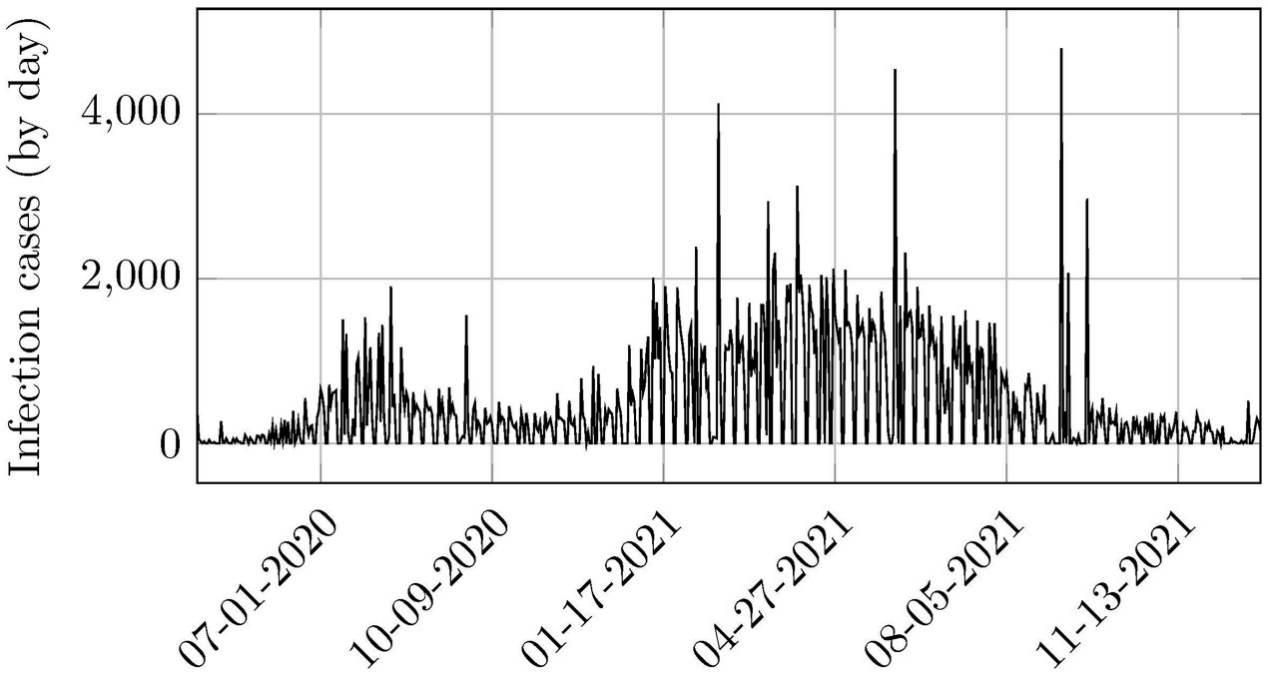
Evolution of COVID-19 infection cases in Belo Horizonte. Source: Belo Horizonte’s Municipal Health (2021).

ARIMA using Google Mobility data time-series was split into two sections: a training set and a test set. For the training set, we used daily data ranging between Apr 20, 2020, to Feb 28, 2021, while the testing set contemplated the period Mar 1, 2021, to Mar 28, 2021. See trajectories in Fig 1.

On the other hand, the time-series database used in the ARIMA model using vaccination coverage data used a different timespan. The time series was divided into two sets. The training set ranged from Mar 2, 2021, to Dec 3, 2021, while the testing set ranged from Dec 4, 2021, to Jul 31, 2021.

### Google mobility reports data

Mobility, in conjunction with vaccination, are important factors to understand the epidemic spreading. Moreover, the use of masks and social vulnerability are other important issues for explaining the incidence of cases in some regions. Here, we are interested in global factors rather than local ones. Thus, the mobility represents a very important issue to this study. For that, there are several possibilities to study its behaviour, among them is the use of Call Detail Records (CDR) collected by mobile phone carriers. However this strategy is controversy, due to privacy problem, and it depends on the mobile operators. In several cities worldwide, the public authorities have used this strategy, however these data are not pubicly available. The other potential source is the use of Google mobility reports data.

Google Community Mobility Reports provides information on the impact of social distancing policies implemented by local authorities upon urban mobility to address the COVID-19 pandemic. They show charts with displacement trends over time by region and across different categories of locations, depicting how visits to places have been changing across different societies. Mobility trends are measured around six types of locations:

- Grocery & pharmacy—places like grocery markets, food warehouses, farmers markets, specialty food shops, drug stores, and pharmacies;
- Parks—places like local parks, national parks, public beaches, marinas, dog parks, plazas, and public gardens;
- Transit stations—places like public transport hubs such as subway, bus, and train stations.
- Retail & recreation—places like restaurants, cafes, shopping centers, theme parks, museums, libraries, and movie theaters;
- Residential—places of residence; and
- Workplaces—places of work.

Google mobility data use the same world-class anonymization technology deployed daily by its products to keep users’ activities private and secure. This includes differentiated privacy, which adds artificial noise to datasets, enabling us to generate insights while avoiding identifying anyone.

These datasets show how visits and length of stay at different places changed compared to a baseline, represented by the median mobility value for any given corresponding day of the week, during the 5-week period: Jan 3 to Feb 6, 2020. The datasets show trends over several months and are generated based on data from users who have opted-in to Location History for their Google Account, so the data represent a sample of Google users. For further details, please refer to: https://www.google.com/covid19/mobility/.

Residence and workplace mobility data were not used in the study as the Coronavirus is more transmissive in public places where the circulation of people is intense. In addition, the Google Mobility data use 2019 as baseline to measure the percentage increase or decrease in mobility. As workers from many companies in Belo Horizonte are still in home-office, one witnessed an emigration of several workers away from the city towards the other municipalities of Minas Gerais State and even to other Brazilian states, a fact that could interfere with model predictions. Moreover, Belo Horizonte is a very important regional center which receives, every day, thousands of persons from its surounding areas. Thus, the number of persons circulating daily in Belo Horizonte is much higher than its population, thus the inclusion of this kind of mobility is recommended, and as the city of Belo Horizonte cannot be treated as a closed system, given the intense circulation therein not only of local dwellers but also inhabitants of nearby municipalities.

**Fig 2:**
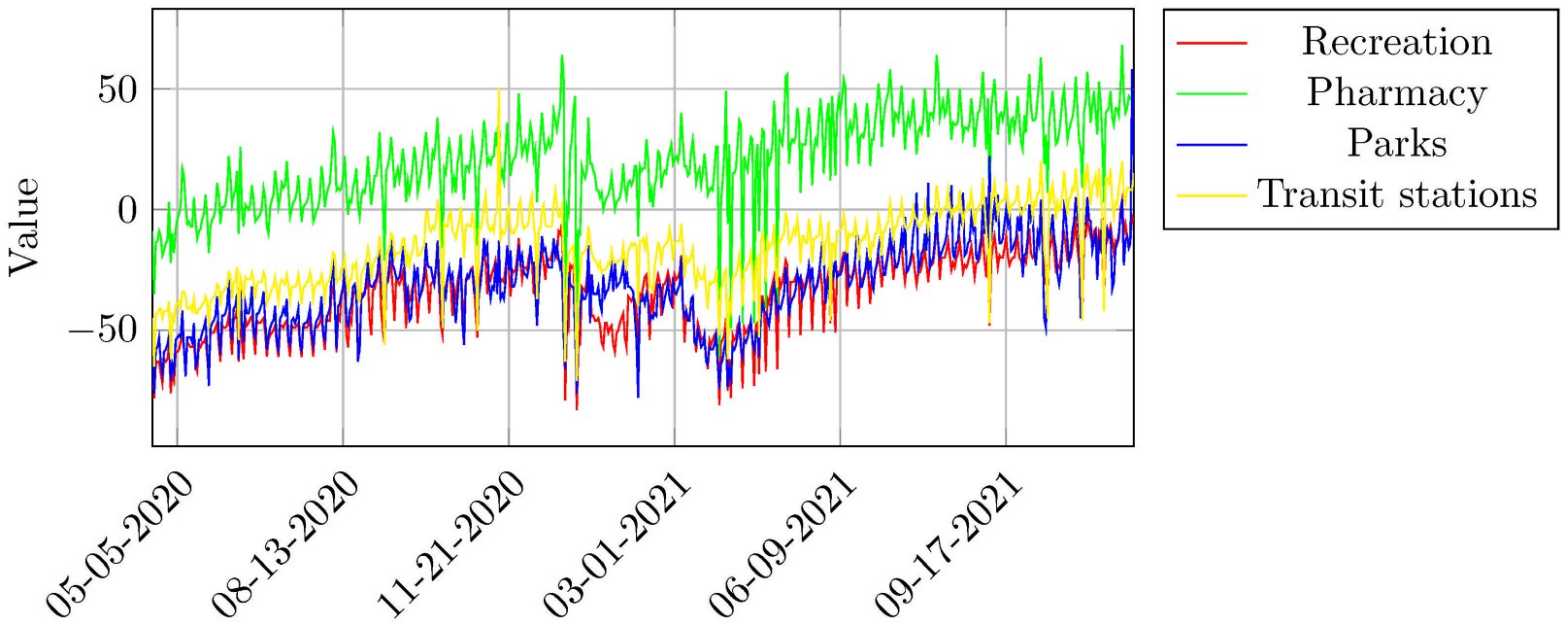
Google mobility reports data for Belo Horizonte. Source: Google Community Mobility Reports (2021).

This study uses Google Mobility Reports data for Belo Horizonte across all the above-mentioned location categories for the time period Apr 20, 2020, to Set 17, 2021. See trajectories in Fig 2.

### Vaccination data

Public health care in Brazil is hierarchically organized and the municipalities are held responsible, among other duties, for the provision of primary health care and immunization campaigns. In the case of the Coronavirus pandemics, COVID-19 vaccines have been purchased by the federal government in large batches and redistributed to the 5,568 municipalities according to their demands.

At the municipal level, COVID-19 vaccine doses have been administered based on a scale of priorities, which initially benefited the health professionals, followed the elderly and those with comorbidities. Vaccination was initiated in Belo Horizonte on Jan 16, 2021, and the local health authorities have been keeping track of immunized and unimmunized populations.

The vaccination data used in this study were provided by Belo Horizonte’s Health Secretariat, and contemplates the cumulative number of doses administered between Jan 25, 2021 and Dec 03, 2021. Four vaccines were used in Belo Horizonte during this period: CoronaVac (Sinovac/Butantan); AstraZeneca (Oxford/Fiocruz); Pfizer (Pfizer/BioNTech) and Janssen (Johnson & Johnson). Complete immunization for these vaccines required at the time the application of two doses, with the following inter-dose intervals: CoronaVac vaccine has an interval of 14 to 28 days; AstraZeneca vaccine has an interval of up to 12 weeks; Pfizer vaccine has an interval of up to 8 weeks; and Janssen is a single-shot vaccine.

**Fig 3:**
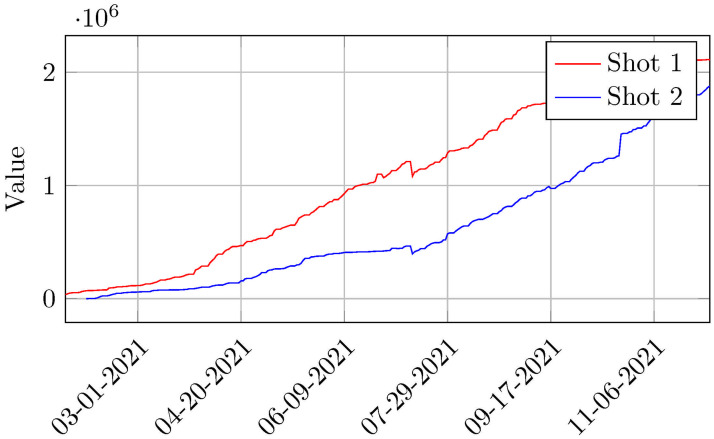
Evolution of first and second shots of COVID-19 vaccination in Belo Horizonte, Brazil (January 25th to December 03rd 2021). Source: Belo Horizonte’s Municipal Health Secretariat (2021).

Fig 3 shows the accumulated number of first and second doses applied per day. Note that the curves show a consistent growth trend, however, the oscillations therein result from the spasmodic way with which the vaccines were made available, either through importation or through domestic manufacture.

Mobility and vaccination are not the sole factors that determinig disease transmission. Other non-pharmaceutical interventions should also be considered in the study of the epidemic spread, such as social distancing, the use of masks, large-scale virus testing, and the degree of dissemination of new variants. However, to the best of our knowledge, no single study has been able to associate all these factors while attempting to predict epidemic spread.

A study estimated the minimum vaccination rate required to avoid the spread of the COVID-19 pandemic, which is 50.91 doses per 100 people [7]. Additionally, a mathematical model suggests that with a mask usage rate of 50% and a 50% effective vaccine, one needs 55–94% vaccination coverage to avoid the exacerbation of the pandemic [8]. In Belo Horizonte, up to Jun 29, 2021, only 20.8% of the target population were vaccinated with a second dose. Therefore, we considered the impact of urban mobility and vaccination to predict future COVID-19 outcomes, although recognizing that other factors may also play a role in the spread of the disease, whose data are unavailable.

### Architecture of the proposed model

In our proposed model, we use the Prophet method (discussed further on) to predict urban mobility, based on social distancing public policy. For this, an exogenous variable (lockdown) was created and received the value 1, when it is within a period of restriction of circulation decreed by the city hall, and receives the value 0, when it falls in the reopening period.

The lockdown dates were Mar 18, 2020; Jun 29, 2020; Jan 11, 2021; and Mar 05, 2021. The reopening dates were May 25, 2020; Jun 08, 2020; Aug 06, 2020; Feb 01, 2021; and Apr 22, 2021. These dates were used to define periods in which the lockdown variable is assigned 0 or 1. After performing the mobility prediction in Prophet, the actual data on the number of cases is joined with the predicted data to be applied as an exogenous variable in the SARIMA model (discussed in the following).

**Fig 4:**
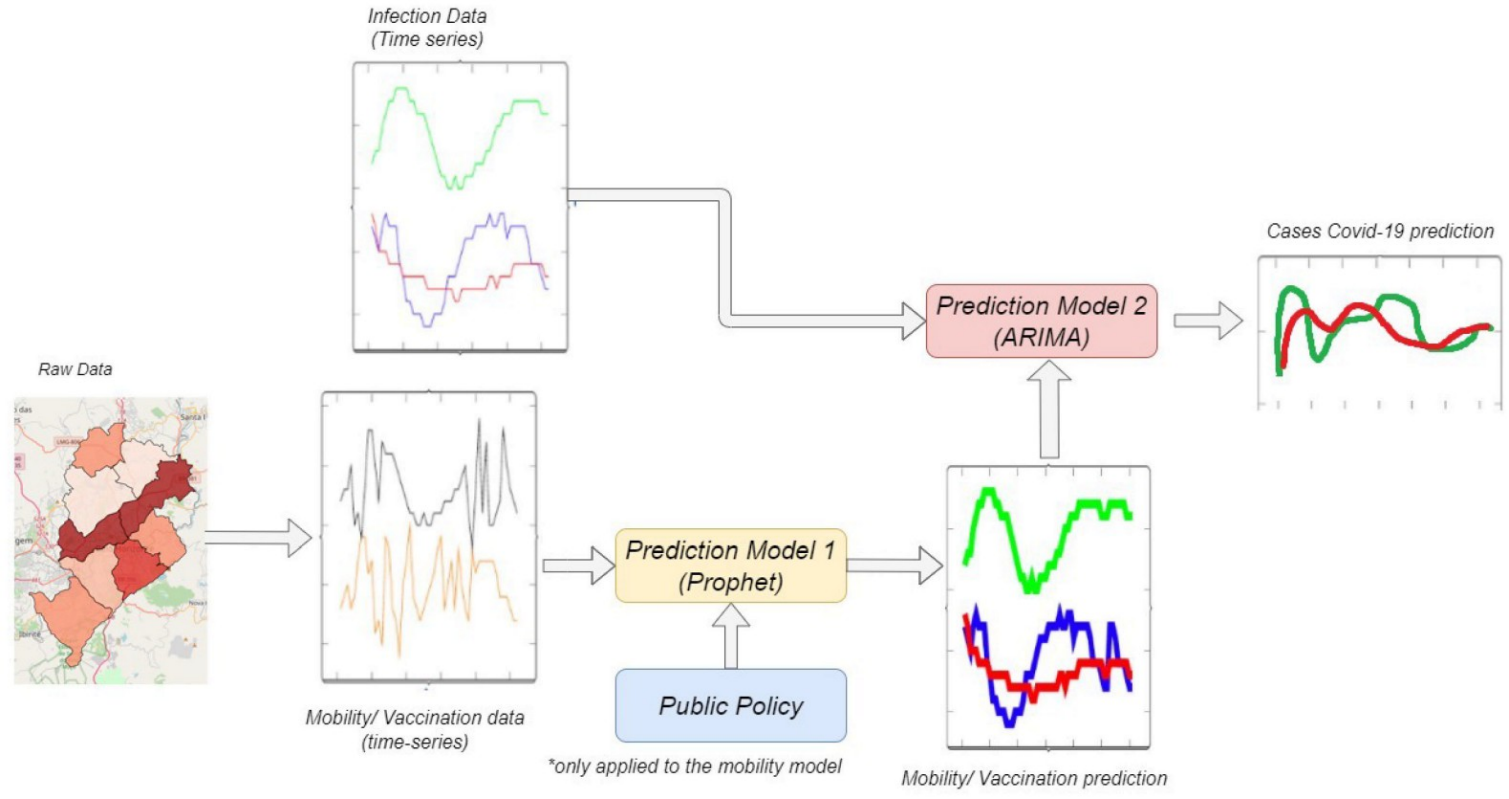
Architectural diagram of the proposed model.

Fig 4 shows the diagram of our proposed model. It is possible to visualize where the data will be applied and in which prediction model it will be used. The SARIMA model receives the training data of the number of cases with the predicted time series in Prophet, and performs the search for the best parameters (p,d,q) according to the smallest AIC value. We also used the auto-arima function in Python to run SARIMA models and the Akaike information criterion (AIC) as reference to identify the best fit model structures spanning out of the stepwise procedure. After finding the best SARIMA model. This proposed model is applied both to vaccination and mobility, however to predict vaccination the lockdown measure was not applied.

Once the best SARIMA models are fitted, they are used to evaluate the relationship between COVID-19 cases and several mobility measures and vaccination data. Model selection was based on the lowest MAE, RMSE and the higher R2. In calculating these metrics values, the prediction results are obtained from the SARIMA model which is built from the training data and the actual data is the data test. The base line of SARIMA modeling will be the SARIMA model with the best results. The best SARIMA models will perform each Google Mobility category as the external variables for the COVID-19 dataset. Similar to the SARIMA process, the best fit combination model is chosen by the lowest RMSE [9].

### Facebook’s prophet algorithm

Prophet is an open-source library developed by Facebook, designed for making forecasts for time series datasets, using the forecasting tools available in Python. The Prophet Algorithm is a model for predicting time-series data. It is based on a decomposition of the time series into three main components: the trend, the seasonality and the holidays. The equation of the Prophet model is the following:

$$\begin{array}{c} y_{t}=g_{t}+s_{t}+h_{t}+\epsilon_{t}, \end{array}$$

where *g*_*t*_ represents the trend, *s*_*t*_ the seasonality and *h*_*t*_ the effect of holidays. *g*_*t*_ is modeled by a piece-wise linear function, *s*_*t*_ by a Fourier series of period *s* (the period of the seasonality in the time series), and *h*_*t*_ by an indicator function multiplied by a Gaussian prior. We use this last component to indicate the days of lockdown during the considered period of time. Prophet is robust event in presence of missing data and trend changes, and usually handles outliers well. Please refer to https://github.com/facebook/prophet for additional information about Prophet [10].

We use Prophet in this work incorporating a Brazilian holiday regressor as a way to minimize the impact of days presenting sudden changes in data, in order to achieve better fitting models.

### ARIMA model

The ARIMA model is a mixture of auto-regressive (AR) and moving average (MA) models, in which both current and historical residual series values in the present time series are linearly expressed [11, 12]. The ARIMA model is an extended version or ARMA, able to deal with non-stationary time series, by differencing the time series until it gets stationnary. The ARIMA model is usually referred to as ARIMA (*p*, *d*, *q*), in which:

- q is the non-seasonal moving average order,
- d is the order of regular differentiation,
- d is the number of differentiation.

The equation of an ARIMA model is the following:

$$\begin{array}{c} y_{t}=\sum_{i=1}^{p}\alpha_{i}\;y_{t-i}+\epsilon_{t}+\sum_{i=1}^{q}\theta_{i}\;\epsilon_{t-i}, \end{array}$$

where the coefficients *α* and *θ* are the parameters of the model and *ϵ* are the error terms.

However, ARIMA does not support seasonal data, or a time series with a repeating cycle. This is not the case with Belo Horizonte’s COVID-19 datagiven the way in which they are gathered, processed and made available by the municipality of Belo Horizonte in weekly bulletins.

Therefore, the Seasonal Autoregressive Integrated Moving Average (SARIMA) or Seasonal ARIMA, is the most appropriate technique. It is an extension of ARIMA that explicitly supports univariate time series data with a seasonal component. This extension considers a new ARIMA model in addition to the classical one. This new model is combined (multiplicatively) to the classical ARIMA model. There are hence three new components: an autoregression (AR), a differencing (I) and moving average (MA) component for the seasonal part of the time series, as well as an additional parameter for the period of the seasonality:

- P is the seasonal order for autoregressive,
- Q is the seasonal order for moving average,
- D is the order of seasonal differentiation and,
- s in the subscription shows the seasonal period.

In the present analysis, for instance, the occurrence of COVID-19 cases varies over the weekly period, thus *s* = 7.

**Fig 5:**
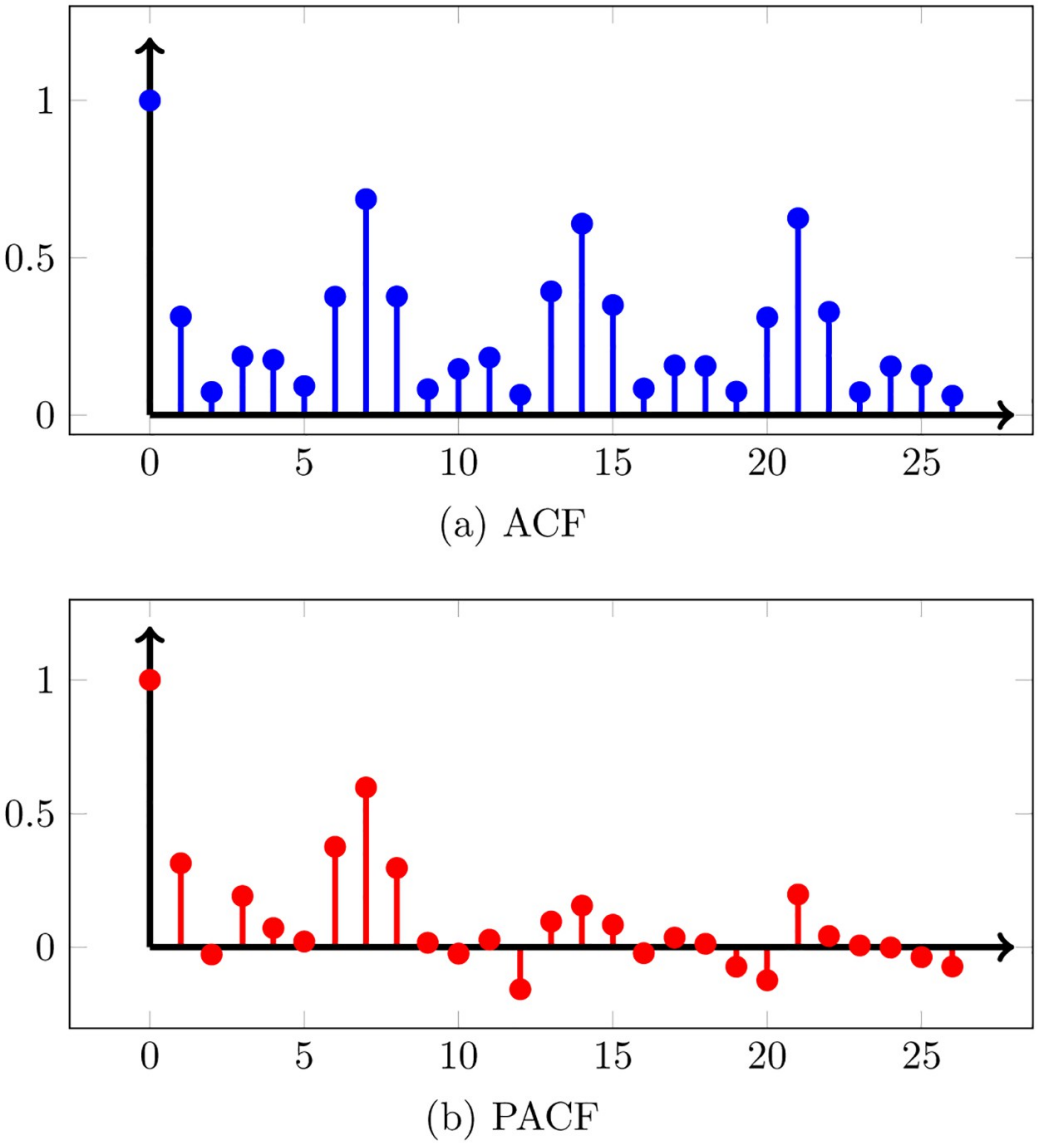
ACF and PACF graphs for COVID-19 cases in Belo Horizonte, Brazil.

The ARIMA family modeling procedure consists of three iterative steps: identification, estimation, and diagnostic checking. Identification is the process of determining seasonal and non-seasonal orders using the autocorrelation functions (ACF) and partial autocorrelation functions (PACF) of the transformed data [13]. The ACF is a statistical tool that measures whether earlier values in the series have some relation to later values. PACF captures the amount of correlation between a variable and a lag of the said variable that is not explained by correlation at all low-order lags.Fig 5 shows the ACF and PACF plots of the time series of the number of COVID-19 cases. Analyzing the ACF and PACF graphs we can obtain candidate values for moving average order (p) and autoregressive (q).

Parameters in the auto SARIMA models are estimated with the conditional least squares (CLS) method [14] after the identification step. The adequacy of the established model for the series is verified by employing white noise tests [15] to check whether the residuals are independent and normally distributed. It is possible that several SARIMA models may be identified, and the selection of an optimum model is necessary. Such selection of models is usually based on the Akaike Information Criterion (AIC) [16]. The *p* and *q* values generated from the ACF and PACF analysis will be the limit combination in making the SARIMA model. Then, by using the COVID-19 dataset, we combine SARIMA’s parameters (p,d,q,P,D,Q) to find the best-fit parameters. Akaike’s Information Criterion (AIC) method is used in selecting the best model by looking at the smallest AIC value using only the training dataset.

We used auto-arima function from *pmdarima* library in Python which fits the best ARIMA model to a univariate time series according to the Akaike information criterion (AIC), which is an estimator of prediction error and thereby relative quality of statistical models for a given set of data [17]. The function performs a stepwise search over possible model and seasonal orders within the constraints provided, and selects the parameters that minimize the given metric.

## Results

Table 1 brings summary statistics for all variables used in the analyses. Notice that between Mar 30, 2021 and Jun 14, 2021, Belo Horizonte displayed on average 551 new COVID-19 cases daily, reaching 4,545 new cases at the height of the pandemic. Mobility trends measured during the same period display different patterns. While mobility around grocery stores and pharmacies have reached an average of 16% above the benchmark, mobility around retail and recreation facilities, as well as around transit stations have significantly decreased compared to the benchmark.

**Table 1:** Summary statistics.

Variables	Min	Max	Mean	Median	St. Dev.
Covid-19 cases	0	4,798	475	218	660
Retail and recreation mobility trends	-83	-6	-39	-36	14
Grocery and pharmacy mobility trends	-75	64	16	17	18
Parks mobility trends	-78	11	-36	-35	14
Transit station mobility trends	-71	50	-19	-18	14

### Prophet model results

**Fig 6:**
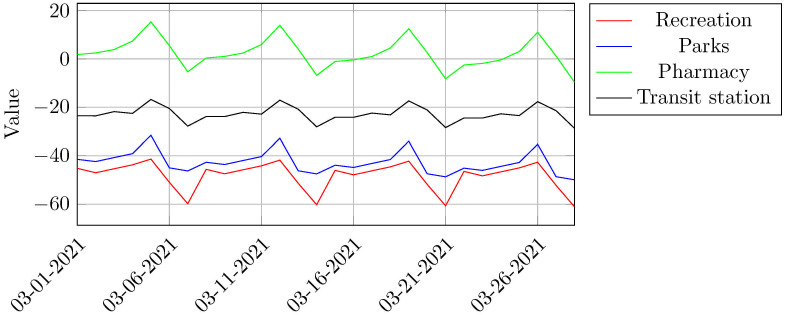
Google mobility prediction data for Belo Horizonte. . Source: Google Community Mobility Reports (2021).

Before predicting COVID-19 infection cases, it is necessary to predict mobility trends in order to provide exogenous information to be input in the infection cases model. This prediction used Prophet framework and Fig 6 brings the results of this procedure.

### SARIMA model results

SARIMA models are fitted to the COVID-19 diseases from Apr 20, 2020 to Dec 31, 20211. Table 2 presents the results of the estimations using the SARIMA processes for the COVID-19 case’s time series. The selection of the best model is performed according to the principle of AIC. Statistical results indicated SARIMA (0,1,2)(1,0,1)[7] as the best fit model, which is applied to forecast future cases.

**Table 2:** COVID-19 auto ARIMA models.

ARIMA (p,d,q)[s]	AIC
ARIMA(0,1,2)(0,0,0)[7]	9,661.274
ARIMA(0,1,3)(0,0,2)[7]	9,528.959
ARIMA(0,1,2)(2,0,0)[7]	9,449.972
ARIMA(0,1,3)(1,0,1)[7]	,9368.011
ARIMA(0,1,2)(1,0,1)[7]	**9,365.655**

Stationary data, along with ACF and PACF, are considered over time. The ACF and PACF correlogram revealed that COVID-19 cases had seasonality impact [18]. The ACF plot shows that sharp significant peak (greater correlation) occurs at lags of 7. This pattern strongly supports the existence of seasonality in the time-series. This is due to the non-recording of data on weekends by the Belo Horizonte city hall. Moreover, time diagram shows that the data are distributed in a horizontal way, the ACF and PACF values decline fairly fast close to zero (Fig 5).

Because the PACF shows a gradual downward trend and the ACF shows sharp cutoffs along with the lags, good candidates for MA, are lag values that have a sharp cutoff (lag = 1, lag = 2). However, it cannot be ruled out that there may be a good model with RA, as there are some sharp cutoffs along with the lags in the ACF.

Fig 6 shows the projections of new cases of COVID-19 with a relative confidence interval of 95% for a four weeks period. The difference between predicted (1,685) and real cases of COVID-19 (2,111) amounts to a 20.18% overestimation, when using exclusively the evolution of COVID-19 cases as reference.

### SARIMAX models results using Google mobility trends data as predictors

Table 3 brings the results of SARIMAX models using the Google Mobility Reports data as external variables. Python’s auto-ARIMA from *pmdarima* algorithms identified the best fit models for each class of mobility data, including the movement around recreation, pharmacy, parks and transit station locales. We have also generated a multivariate ARIMA using all mobility measures in order to evaluate the combined effects of intraurban movement upon COVID-19 cases. The cumulative confirmed COVID-19 cases in 28 days turned out to be 27,666.

**Table 3:** ARIMA models results.

EXTERNAL VARIABLES	ARIMA (p,d,q)(P,D,Q)[7]	AIC	28-day projection	MAE	RMSE	MSE	R² Score
Retail and recreation mobility trends	(3,1,1)x(1,0,1)	4,628.504	22,821.889	**429.900**	621.497	386,258.768	0.412732
Grocery and pharmacy mobility trends	(1,1,2)x(1,0,1)	4,627.377	**27,435.943**	434.513	**579.207**	**335,481.495**	**0.489934**
Parks mobility trends	(0,1,1)x(1,0,3)	4631.657	22711.576	480.939	664.034	440,941.656	0.329592
Transit station mobility trends	(1,1,2)x(1,0,1)	**4,625.756**	32,244.847	485.734	598.838	358,607.271	0.454773
Multivariate	(0,1,2)x(1,0,1)	4,634.617	28,384.627	491.513	629.845	396,704.807	0.396850

**Fig 7:**
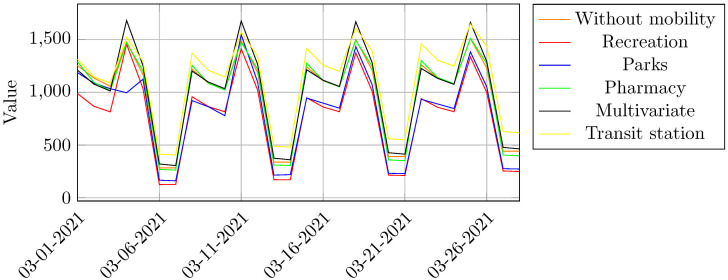
Prediction of COVID-19 infection cases taking into account mobility issues.

Curiously, the multivariate model is the one presenting the highest AIC value, frustrating the expectations of a better performance. As Google Mobility Reports data are based on intraurban travel behavior of Google Maps users, mobility data are not independently generated, as one individual may carry his/her cell phone in a shopping trip along which he/she passes by pharmacies, parks, transit stations and recreation facilities (Table 3). Thus, multicollinearity among these variables is a serious issue, compromising the multivariate model’s performance. On the other hand, single external factors ARIMA models performed much better, with mobility around grocery/pharmacy and transit station displaying the lowest AIC value. Model results are in fine tune with the reality of Belo Horizonte. Pharmacies were not closed during the lockdown period, on the contrary, they functioned as a thermometer to know if the population was getting sick. Interestingly, when the number of cases reached peaks, that of pharmacy mobility also increased. On the other hand, mobility around transit stations is also an important predictor of COVID-19 cases, as despite the social distancing measures decreed by Belo Horizonte mayor’s office, several economic sectors remained active. Therefore, commuting journeys between home and place of work, captured by Google mobility data, significantly contributed to the spread of Coronavirus. In Fig 7, the prediction of infection cases taking into account different Google mobility analysis are showed.

Another way of examining model performance is through the metrics MAE, MSE, RMSE and *R*^2^. MAE (Mean absolute error) represents the difference between the original and predicted values extracted by averaged the absolute difference over the data set. It measures the average of the residuals in the dataset. MAE is less sensitive to outliers compared to RMSE. MSE (Mean Squared Error) measures the average of the squares of the errors. RMSE (Root Mean Squared Error) is the error rate by the square root of MSE. It measures the standard deviation of residuals. RMSE represents the estimated white noise standard deviation in ARIMA analysis, and can only be compared between models whose errors are measured in the same units. The lower the MSE and RMSE, the better the model. *R*^2^ (Coefficient of determination) represents the coefficient of how well the values fit compared to the original values. The value from 0 to 1 interpreted as percentages. The higher the value, the better the model. is [19]. From the measures illustrated in Table 3, we can say that MSE, RMSE and *R*^2^ present consistent behaviour, confirming the validity of our results.

### SARIMAX model results using vaccination as predictor

Table 4 shows the prediction of COVID-19 cases using vaccination data and the forecast of COVID-19 applying a mix of independent variables (4 types of mobility + vaccination). Notice that the model with all variables had the smallest number of predicted cases compared to the model that exclusively incorporated vaccination data. We can argue that when vaccination advances, there is a higher correlation between both vaccination and mobility data, which can explain the greater approximation between predicted and real cases.

**Table 4:** ARIMA model results taking into account vaccination as predictor.

VARIABLE	ARIMA (p,d,q)(P,D,Q)[s]	AIC	56-day projection	Real cumulative confirmed cases
Vaccination	ARIMA(4,1,2)(6,0,0)[7]	**3,905.451**	9,611.7	5,904
Vaccination + Mobility	ARIMA(2,1,2)(2,0,2)[7]	8,651.442	**5,770.5**	5,904

**Fig 8:**
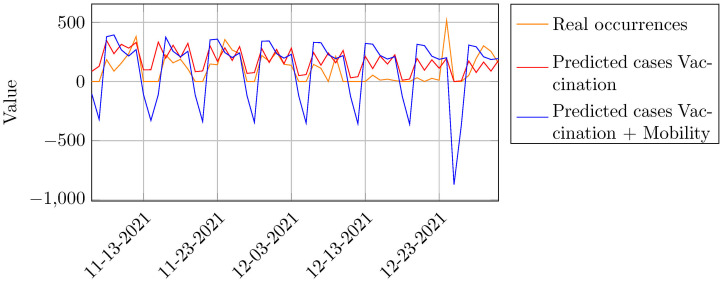
Prediction of COVID-19 infection cases taking into vaccination issues.

Fig 8 shows the comparison between the models applying the variables as shown in Table 4. The multivariate model presents seasonal patterns analogous to the vaccination one, although the waves have greater amplitudes, which can be explained by the increase of mobility as vaccination progresses.

In Tables 5–9, we illustrated the statistical methods obtained by the proposed models when different strategies for computing the Google mobility data are employed.

**Table 5:** SARIMA Model recreation results.

SARIMAX(3,1,1)x(1,0,1,7)						
AIC: 4628.504						
parameters	coef	sdt err	z	P>‖*z*‖	[0.025	0.975]
**x1**	4.1653	2.695	1.546	0.122	-1.117	9.447
**ar.L1**	-0.1751	0.075	-2.341	0.019	-0.322	-0.028
**ar.L2**	-0.1411	0.074	-1.910	0.056	-0.286	0.004
**ar.L3**	-0.2234	0.058	-3.856	0.000	-0.337	-0.110
**ma.L1**	-0.8196	0.040	-20.274	0.000	-0.899	-0.740
**ar.S.L7**	0.9701	0.034	28.801	0.000	0.904	1.036
**ma.S.L7**	-0.7615	0.070	-10.920	0.000	-0.898	-0.625
**sigma2**	1.374e+05	5647.957	24.328	0.000	1.26e+05	1.48e+05
**Ljung-Box (L1) (Q):**			0.37	**Jarque-Bera (JB):**		8671.94
**Prob(Q):**			0.54	**Prob(JB):**		0.00
**Heteroskedasticity (H):**			4.41	**Skew:**		2.85
**Prob(H) (two-sided):**			0.00	**Kurtosis:**		28.10

**Table 6:** SARIMA Model pharmacy results.

SARIMAX(1,1,2)x(1,0,1,7)						
AIC: 4627.377						
parameters	coef	sdt err	z	P>—*z*—	[0.025	0.975]
**x1**	3.5709	2.047	1.744	0.081	-0.442	7.584
**ar.L1**	0.6597	0.104	6.356	0.000	0.456	0.863
**ma.L1**	-1.7428	0.086	-20.312	0.000	-1.911	-1.575
**ma.L2**	0.7976	0.075	10.694	0.000	0.651	0.944
**ar.S.L7**	0.9808	0.027	35.689	0.000	0.927	1.035
**ma.S.L7**	-0.8044	0.065	-12.327	0.000	-0.932	-0.677
**sigma2**	1.44e+05	6345.621	22.686	0.000	1.32e+05	1.56e+05
**Ljung-Box (L1) (Q):**			0.69	**Jarque-Bera (JB):**		9546.61
**Prob(Q):**			0.40	**Prob(JB):**		0.00
**Heteroskedasticity (H):**			4.55	**Skew:**		2.86
**Prob(H) (two-sided):**			0.00	**Kurtosis:**		29.40

**Table 7:** SARIMA Model park results.

SARIMAX(0,1,1)x(1,0,3,7)						
AIC: 4631.657						
parameters	coef	sdt err	z	P>‖*z*‖	[0.025	0.975]
**x1**	3.5325	3.545	0.997	0.319	-3.415	10.480
**ma.L1**	-0.9329	20.019	-49.161	20.000	-0.970	-0.896
**ar.S.L7**	0.9202	0.056	16.412	0.000	0.810	1.030
**ma.S.L7**	-0.6165	0.082	-7.524	0.000	-0.777	-0.456
**ma.S.L14**	-0.1649	0.081	-2.028	0.043	-0.324	-0.006
**ma.S.L21**	0.1547	0.065	2.364	0.018	0.026	0.283
**sigma2**	1.399e+05	5462.068	25.609	0.000	1.29e+05	1.51e+05
**Ljung-Box (L1) (Q):**			1.11	**Jarque-Bera (JB):**		8174.44
**Prob(Q):**			0.29	**Prob(JB):**		0.00
**Heteroskedasticity (H):**			4.40	**Skew:**		2.89
**Prob(H) (two-sided):**			0.00	**Kurtosis:**		27.32

**Table 8:** SARIMA Model transit station results.

SARIMAX(1,1,2)x(1,0,1,7)						
AIC: 4625.756						
parameters	coef	sdt err	z	P>‖*z*‖	[0.025	0.975]
**x1**	8.4167	2.345	3.589	0.000	3.821	13.013
**ar.L1**	0.8097	0.044	18.313	0.000	0.723	0.896
**ma.L1**	-1.8941	0.030	-62.415	0.000	-1.954	-1.835
**ma.L2**	0.9362	0.027	34.680	0.000	0.883	0.989
**ar.S.L7**	0.9828	0.030	33.216	0.000	0.925	1.041
**ma.S.L7**	-0.8336	0.074	-11.243	0.000	-0.979	-0.688
**sigma2**	1.604e+05	9605.464	16.698	0.000	1.46e+05	1.79e+05
**Ljung-Box (L1) (Q):**			0.45	**Jarque-Bera (JB):**		8545.17
**Prob(Q):**			0.50	**Prob(JB):**		0.00
**Heteroskedasticity (H):**			4.11	**Skew:**		2.72
**Prob(H) (two-sided):**			0.00	**Kurtosis:**		27.93

**Table 9:** SARIMA Model multivariable results.

SARIMAX(0,1,2)x(1,0,1,7)						
AIC: 4634.617						
parameters	coef	sdt err	z	P>‖*z*‖	[0.025	0.975]
**x1**	-16.8061	6.336	-2.653	0.008	-29.224	-4.388
**x2**	4.2909	4.215	1.018	0.309	-3.970	12.551
**x3**	0.5806	4.717	0.123	0.902	-8.665	9.827
**x4**	13.3098	4.343	3.065	0.002	4.799	21.821
**ma.L1**	-1.0824	0.068	-15.898	0.000	-1.216	-0.949
**ma.L2**	0.1362	0.068	2.015	0.044	0.004	0.269
**ar.S.L7**	0.9596	0.040	24.070	0.000	0.881	1.038
**ma.S.L7**	-0.6916	0.094	-7.367	0.000	-0.876	-0.508
**sigma2**	1.706e+05	8257.039	20.658	0.000	1.54e+05	1.87e+05
**Ljung-Box (L1) (Q):**			0.06	**Jarque-Bera (JB):**		10439.29
**Prob(Q):**			0.80	**Prob(JB):**		0.00
**Heteroskedasticity (H):**			4.44	**Skew:**		3.12
**Prob(H) (two-sided):**			0.00	**Kurtosis:**		30.55

## Conclusion and future work

By analyzing the mobility graph and transposing it with the graph of the number of real cases, it is possible to infer that there was an increase in the number of cases with increasing mobility levels. The projection of the number of cases with each Google mobility variable has similar seasonal characteristics, the difference being the number of cases. In addition, the grocery/pharmacy application had the smallest error in the model (absolute error of 0.835%) about the accumulated cases: 27,666 real against 27,435 predicted cases.

We can highlight that using the model with the combination of mobility and vaccination, even with a larger projection, the error metrics were smaller compared to the mobility model. Besides, we managed to find a projection value very close to the real value, 5,904 real cases accumulated, against 5,770 of foreseen cases. Moreover, the model using vaccination as a dependent variable turned out to be weaker, with 9,611 predicted cases. Still, this model recognizes well the seasonality pattern of the series. A hypothesis for this result is that when the vaccination progresses, the number of cases decreases, and consequently, the population resume its normal life by increasing the urban mobility. Thus, thanks to the high vaccination rates, even after mobility has been with higher mobility, the impact of new cases is smaller.

In addition, the mobility variable starts to lose importance since even after mobility has been fully reestablished in Belo Horizonte, the number of cases continued to fall. Finally, the proposed model can be replicated, as long as city level COVID-19 and Google Mobility Reports datasets are available.

In terms of limitations, in this study, we have studied the statistical associations between the flow of people in Belo Horizonte, by using urban mobility from Google Mobility reports data, and the occurrence of cases in different periods, considering public interventions to limit activities in the city in conjunction with the vaccination progresses. Although these results already contribute to a better understanding of these relationships, in future studies we will try to identify and understand the mechanisms involved in these associations in order to establish and test the effectiveness of interventions.

This work was restricted to the application of the Prophet and ARIMA methods, however, there are other ways of performing time-series predictions, such as the LSTM (Long short-term memory), artificial recurrent neural network architecture used in the field of deep learning, but that could also be applied to time series. Another method that could be applied is the SIR model, which is specifically aimed at predicting communicable diseases, applying three ordinary differential equations for susceptible, infectious, and recovered numbers of people.

## Supporting information

S1 File (ZIP)
